# Membranous nephropathy: Clearer pathology and mechanisms identify potential strategies for treatment

**DOI:** 10.3389/fimmu.2022.1036249

**Published:** 2022-11-02

**Authors:** Edmund Y. M. Chung, Yuan M. Wang, Karen Keung, Min Hu, Hugh McCarthy, Germaine Wong, Lukas Kairaitis, Bhadran Bose, David C. H. Harris, Stephen I. Alexander

**Affiliations:** ^1^ Centre for Kidney Research, The Children’s Hospital at Westmead, Westmead, NSW, Australia; ^2^ Department of Nephrology, Prince of Wales Hospital, Randwick, NSW, Australia; ^3^ The Centre for Transplant and Renal Research, Westmead Institute of Medical Research, Westmead, NSW, Australia; ^4^ Department of Nephrology, The Children’s Hospital at Westmead, Westmead, NSW, Australia; ^5^ Department of Nephrology, Sydney Children’s Hospital, Randwick, NSW, Australia; ^6^ Department of Nephrology, Westmead Hospital, Westmead, NSW, Australia; ^7^ Department of Nephrology, Blacktown Hospital, Blacktown, NSW, Australia; ^8^ Department of Nephrology, Nepean Hospital, Kingswood, NSW, Australia

**Keywords:** membranous nephropathy, Heymann nephritis, autoantibody, immunology, podocyte, treatment

## Abstract

Primary membranous nephropathy (PMN) is one of the common causes of adult-onset nephrotic syndrome and is characterized by autoantibodies against podocyte antigens causing *in situ* immune complex deposition. Much of our understanding of the disease mechanisms underpinning this kidney-limited autoimmune disease originally came from studies of Heymann nephritis, a rat model of PMN, where autoantibodies against megalin produced a similar disease phenotype though megalin is not implicated in human disease. In PMN, the major target antigen was identified to be M-type phospholipase A2 receptor 1 (PLA2R) in 2009. Further utilization of mass spectrometry on immunoprecipitated glomerular extracts and laser micro dissected glomeruli has allowed the rapid discovery of other antigens (thrombospondin type-1 domain-containing protein 7A, neural epidermal growth factor-like 1 protein, semaphorin 3B, protocadherin 7, high temperature requirement A serine peptidase 1, netrin G1) targeted by autoantibodies in PMN. Despite these major advances in our understanding of the pathophysiology of PMN, treatments remain non-specific, often ineffective, or toxic. In this review, we summarize our current understanding of the immune mechanisms driving PMN from animal models and clinical studies, and the implications on the development of future targeted therapeutic strategies.

## Introduction

Primary membranous nephropathy (PMN) is one of the commonest causes of adult-onset nephrotic syndrome with an incidence of 12 cases per million per year (1). It is a kidney-limited autoimmune disease characterized by *in situ* formation of subepithelial glomerular immune deposits containing immunoglobulin G (IgG) autoantibodies and complement (2). While spontaneous disease remission occurs in around 32% of patients, approximately a third ultimately progress to kidney failure needing dialysis or kidney transplantation (1). The podocyte antigens responsible for eliciting an autoimmune response in PMN remained elusive until 2009 when M-type phospholipase A2 receptor 1 (PLA2R) was discovered, followed by thrombospondin type-1 domain-containing protein 7A (THSD7A) in 2014 (3, 4). These discoveries improved the diagnosis of PMN with anti-PLA2R autoantibody testing having a specificity of 99%, which together with the kidney biopsy light microscopy findings of subepithelial “spikes” of the glomerular basement membrane (GBM) on silver methenamine stain, immunofluorescence finding of granular deposits of IgG along the glomerular capillary wall, and electron microscopy finding of exclusive subepithelial localization of electron-dense deposits, differentiate PMN from other forms of nephrotic syndrome (1, 5). However, despite this improved understanding, treatments for PMN remain non-specific. Cytotoxic therapy (chlorambucil or cyclophosphamide) plus glucocorticoids lower the 10-year incidence of kidney failure to approximately 10% (compared to 40% in those receiving supportive treatment) but is associated with significant toxicity such as infertility, infection and malignancy (6–10). Calcineurin inhibitors have proven short-term efficacy though are limited by high relapse rate of 40% and long-term nephrotoxicity (11). The anti-CD20 monoclonal antibody rituximab targeting B cells is the most targeted treatment to date but may not eliminate autoreactive B cells due to changes in the CD20 antigen, does not eliminate high-affinity antibody-producing plasma cells that do not express CD20, and fails to induce complete or partial remission in up to 40% of patients (12, 13). In comparing these existing treatments, a recent trial found cyclophosphamide plus glucocorticoids to be superior to calcineurin inhibitors plus single-dose rituximab (total 1g) though there was a higher anti-PLA2R autoantibody titer at baseline in the calcineurin inhibitor/rituximab group, which together with the low dose of rituximab used, may have contributed to worse outcomes (14). In contrast, rituximab (total 3g) was superior to calcineurin inhibitors (12), but rituximab (total 2g) was comparable to cyclophosphamide plus glucocorticoids (15). Lastly, recurrent membranous nephropathy (MN) after kidney transplantation also occurs in 35-50% of patients and accounts for 50% allograft loss 5 years after diagnosis, highlighting the unmet need for targeted treatments in PMN (16–19).

Antigen-independent treatments targeting autoantibody production is attractive and feasible given the multitude of disease antigens implicated in PMN particularly with improved antibodies targeting plasma cells. Targeted treatments against antigen-specific B cells or antigen-specific T cells helpers directed against antigens linked to PMN, using chimeric antigen receptor (CAR) T cells, chimeric autoantigen receptor expressing (CAAR) T cells, or CAR regulatory T cells (Tregs) to eliminate or suppress antigen-specific effectors are also conceivable. However, these experimental treatments remain untested in human autoimmune disease and rigorous testing in animal models of PMN is required (20). In this review, we compare the immune mechanisms driving PMN and associated animal models, and implications for evaluating novel targeted therapeutics.

## Pathological features in animal models of membranous nephropathy

### Heymann nephritis

Heymann et al. described active Heymann nephritis (AHN) in 1959, where immunization of the proximal tubular antigen Fx1A with complete Freund’s adjuvant in Lewis rats caused development of anti-Fx1A antibodies and *in situ* glomerular subepithelial immune complex formation after 4 weeks, and proteinuria after 8-10 weeks (21). Passive Heymann nephritis (PHN) is a more limited model induced by administration of sheep anti-rat Fx1A antibodies with cell- and blood-free perfusion to prevent immune complex formation in the circulation (22, 23). While PHN was widely used to study the effector mechanisms caused by glomerular IgG deposition, it does not recapitulate autologous formation of podocyte autoantibodies seen in AHN and which leads to PMN.

### Passive administration of anti-PLA2R or anti-THSD7A antibodies in mice

The identification of antigens implicated in PMN has led to the development of mouse models where passive administration of anti-PLA2R and anti-THSD7A antibodies into transgenic mice with murine full-length PLA2R on podocytes and wild-type mice respectively were able to induce similar disease to PMN with subepithelial glomerular IgG deposition, C3 deposition and proteinuria (24–27).

### Transgenic mice expressing human PLA2R specifically on podocytes

Recently, Tomas et al. have described transgenic knock-in mice expressing human PLA2R specifically on podocytes. These mice spontaneously developed anti-human PLA2R autoantibodies after 3 weeks, nephrotic syndrome with glomerular deposition of murine IgG1 (equivalent of human IgG4) and complement activation after 4 weeks, and glomerular subepithelial electron-dense deposits after 6 weeks (28). Overall, this model faithfully recapitulates human PMN.

### Passive administration of anti-aminopeptidase A, anti-dipeptidyl peptidase IV, or anti-podocyte antibodies in rats or mice

Passive administration of antibodies against mouse aminopeptidase A (29, 30), dipeptidyl peptidase IV (31), and anti-mouse podocyte antibodies were also able to induce glomerular IgG deposition with proteinuria but without complement activation, unlike human PMN (32, 33). Furthermore, these antigens are not implicated in human PMN.

### Exogenous antigen deposition onto the glomerular basement membrane in rabbits or mice

Intravenous injections of cationic bovine serum albumin (BSA) and immunization of recombinant human non-collagenous domain 1 of α3(IV) collagen (rh-α3NC1) both produced significant albuminuria and subepithelial deposits of IgG and C3 (34, 35). However, glomerular deposition of exogenous antigen (BSA and rh-α3NC1) in these models significantly differs from the pathogenesis of human PMN (35).

## Pathological features in human membranous nephropathy

Human PMN is characterized by heterogeneous presentation and clinical course with around a third of patients undergoing spontaneous remission, a third experiencing persistent proteinuria, and a third progressing to kidney failure (36). While light microscopy findings on kidney biopsy also exhibit some variability, ranging from a normal appearance early in disease to thickened GBM as disease progresses, granular deposition of IgG and C3 in glomeruli on immunofluorescence and electron-dense subepithelial deposits on electron microscopy are seen at any stage of disease (1, 36).

## Antigens and autoantibodies in animal models of membranous nephropathy

### Heymann nephritis

The pathogenic antigen in Fx1A was identified as a 330-kDa glycoprotein called megalin, which is a low-density lipoprotein receptor involved in protein endocytosis and transcytosis (**Table 1**) (53–56). Another autoantibody in AHN was subsequently identified targeting the 44-kDa receptor associated protein (RAP), which acts as an endoplasmic reticulum chaperone for megalin (55).

**Table 1:** Disease antigens implicated in Heymann nephritis and membranous nephropathy.

Antigen	Normal tissue expression (References)	Function of antigen	Prevalence of antigen in disease	IgG subclass deposited in glomeruli	Serum antibody	Pathogenicity of antibody	Complement activation	Mean age (years)	Sex (M:F)	Other disease associations
Heymann nephritis										
Megalin (in Fx1A)	Podocyte, proximal tubular brush border, type II pneumocytes, epididymis (37, 38)	Low-density lipoprotein receptor, calcium handling, mediates transcytosis of certain proteins (eg. albumin, retinol binding protein) and degradation of other proteins in lysosomes	100%	Rat IgG2b^a^ >> Rat IgG1^b^	Yes	Yes	C3, C5b-9	NA	NA	Human ABBA disease
RAP	All cells, primarily in kidney and brain (39)	Endoplasmic reticulum chaperone for lipid receptors such as megalin	Unclear	Unclear	Yes	Yes	Unclear	NA	NA	None
Primary membranous nephropathy										
PLA2R	Podocyte (3)	Unclear in kidney. Regulates cellular senescence, migration, hormone and cytokine release	70%	IgG4	Yes (non-reducing)	Highly likely	C3, C5b-9, MBL	48	2:1	None
THSD7A	Podocyte (4)	Stabilizes and enhances podocyte adhesion	2.5%	IgG4	Yes (non-reducing)	Yes	C5b-9, MBL,^c^ C3b^c^	50	1:3	Malignancy (33%)
NELL1	Osteoblast, brain, kidney tubule (40)	Unclear in kidney	2%^d^	IgG1	Yes (non-reducing)	Unclear	C3, rarely C1q	63	1:1	Malignancy (33%)
Sema3B	Most organs (especially CNS), kidney tubule, endothelial cells, podocyte (41, 42)	Unclear in kidney. CNS development	Unclear^e^	IgG1 >> IgG4	Yes (reducing)	Unclear	C3, rarely C1q	15	3:2	None
PCDH7	Brain, kidney tubule (43)	Unclear in kidney. Cell signaling	2%	IgG4 >> IgG1, IgG2, IgG3	Yes (non-reducing)	Unclear	Trace C3	61	3:1	Autoimmune disease (21%), malignancy (14%)
Serine protease HTRA1	Non-specific, podocyte (44)	Unclear in kidney. Cell growth, apoptosis, and inflammation	1-2%	IgG4	Yes (non-reducing or reducing)	Unclear	C3, rarely C1q	67	1:1	None
NTNG1	Brain, podocyte (45)	Unclear in kidney. Axonal adhesion molecule	0.4%	IgG4	Yes (non-reducing)	Unclear	Unclear	58	Male in all 3 cases	None
Secondary membranous nephropathy										
EXT1/EXT2	Non-specific, podocyte in kidney (46)	Synthesize heparan-sulfate backbone of glycosaminoglycan residues on proteins	28% MLN	IgG1	No	Unclear	C3, C1q	36	1:4	Autoimmune disease (71%), malignancy (8%)
NCAM1	CNS, thyroid, adrenal, heart, stomach, immune cells, kidney interstitial cells, podocyte (47)	Unclear in kidney. Synaptic plasticity, neuronal migration, axonal branching, fasciculation and synaptogenesis in CNS	7% MLN	IgG1 > IgG3 > IgG2, IgG4	Yes (non-reducing)	Unclear	C3, C1q	34	1:2	Autoimmune disease, especially SLE (90%)
TGFBR3	Podocyte, mesangial cell, endothelial cell (48)	Accessory receptor for TGF-β signaling, protecting against autoimmunity	6% MLN	IgG1, IgG2 or IgG3	No	Unclear	C3, C1q	40	1:16	Autoimmune disease, especially SLE (82%)
CNTN1	CNS, PNS, kidney glomeruli (49)	Unclear in kidney. Neuronal cell adhesion molecule that regulates myelination and nodal/paranodal organization in CNS	Unclear	IgG4	Yes	Unclear^f^	C3	70	Male in all 5 cases	CIDP (100%)
FAT1	Podocyte, kidney proximal tubular epithelial cells, CNS, skin, esophagus, lung (50)	Unclear in kidney. Mediates calcium-dependent cell-cell recognition and adhesion	Unclear	IgG4	Yes (non-reducing or reducing)	Unclear	Mild C3 (0-1+)	60	9:5	Allogeneic HSCT (100%)
Neonatal membranous nephropathy										
NEP	Podocyte, lung, intestine, adrenal, prostate, breast, uterus, CNS (51, 52)	Regulate levels of various peptides (eg. atrial natriuretic peptide)	Unclear	IgG1	Yes	Yes	C3, C1q	From birth	1:1	None

RAP, receptor associated protein; NA, not applicable; ABBA, kidney anti brush border antibodies and kidney failure; PLA2R, M-type phospholipase A2 receptor 1; MBL, mannan-binding lectin; THSD7A, thrombospondin type-1 domain-containing protein 7A; NELL1, Neural epidermal growth factor-like 1 protein; Sema3B, Semaphorin 3B; CNS, central nervous system; PCDH7, Protocadherin 7; SLE, systemic lupus erythematosus; HTRA1, high temperature requirement A serine peptidase 1; NTNG1, netrin G1; EXT1/EXT2, exostosin 1/exostosin 2; MLN, membranous lupus nephritis; NCAM1, neural cell adhesion molecule 1; SLE, systemic lupus erythematosus; TGFBR3, type III transforming growth factor β receptor; CNTN1, contactin 1; PNS, peripheral nervous system; CIDP, chronic inflammatory demyelinating polyneuropathy; FAT1, protocadherin FAT1; HSCT, hematopoietic stem cell transplant; NEP, neutral endopeptidase.

a Rat IgG2b equivalent to human IgG1.

b Rat IgG1 equivalent to human IgG4.

c Only in experimental data of murine glomeruli exposed to human sera containing anti-THSD7A antibodies.

d Prevalence derived from “Wang et al. Neural Epidermal Growth Factor–Like 1 Protein–Positive Membranous Nephropathy in Chinese Patients. Clin J Am Soc Nephrol. 2021 May 8;16(5):727-35” since discovery cohort only reported prevalence of NELL1-associated PMN of 16% in PLA2R-negative PMN.

e Discovery cohort only reported prevalence of Sema3B-associated PMN of 9% in PLA2R-, THSD7A-, EXT1/EXT2-, and NELL-1-negative PMN.

f Anti-CNTN1 proven to be pathogenic in CIDP but not MN.

**Figure 1:**
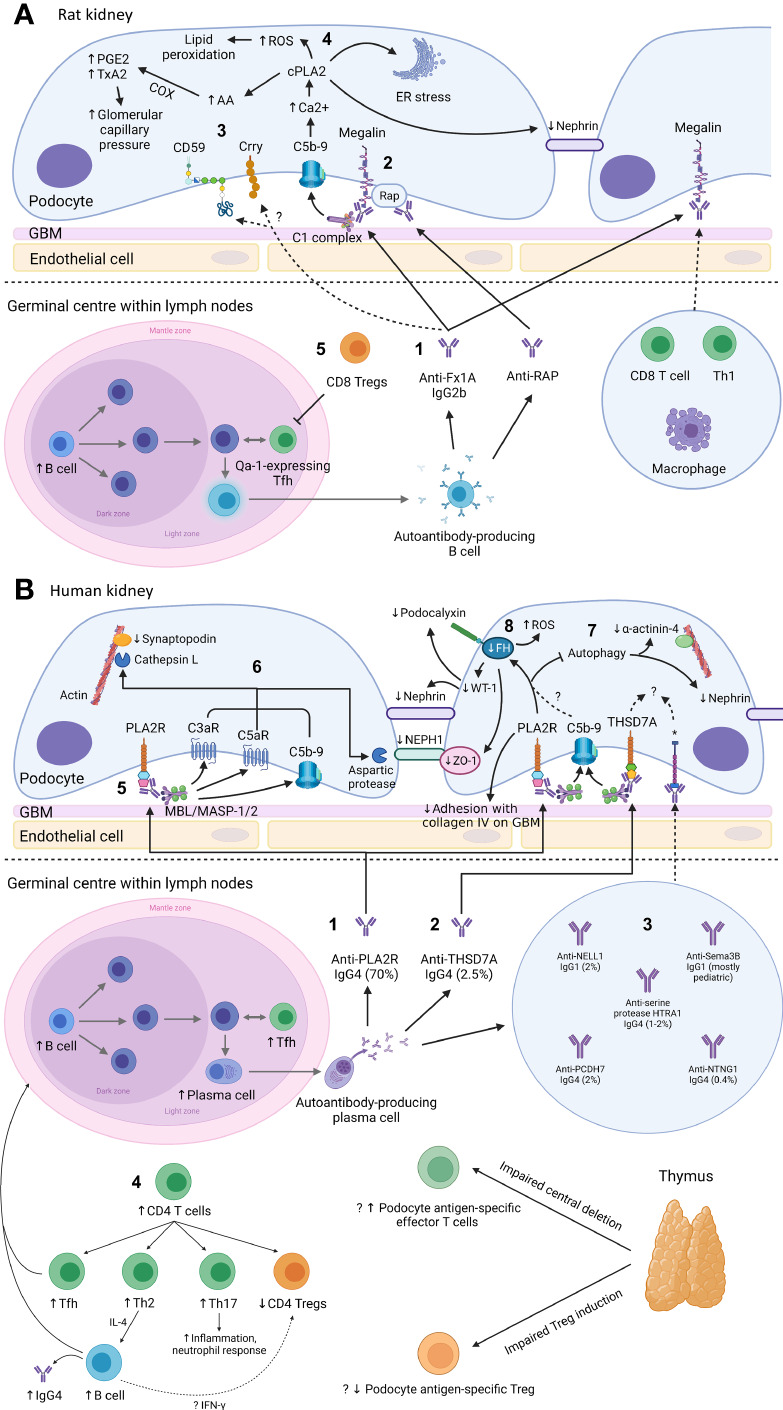
Mechanism of podocyte injury in Heymann nephritis **(A)** and primary membranous nephropathy **(B)** Created with BioRender.com. GBM, glomerular basement membrane; ROS, reactive oxygen species; cPLA2, cytosolic phospholipase A2; PGE2, prostaglandin E2; TxA2, thromboxane A2; COX, cyclo-oxygenase; AA, arachidonic acid; ER, endoplasmic reticulum; RAP, receptor associated protein; Treg, regulatory T cell; Th1, CD4^+^ T helper-1 cell; Tfh, CD4^+^ T follicular helper cell; FH, fumarate hydratase; WT-1, Wilms tumor-1; PLA2R, M-type phospholipase A2 receptor 1; C3aR, C3a receptor; C5aR, C5a receptor; MBL, mannan-binding lectin; MASP, mannan-binding lectin serine protease; ZO-1, zonula occludens-1; THSD7A, thrombospondin type-1 domain-containing protein 7A; NELL1, neural epidermal growth factor-like 1 protein; Sema3B, semaphorin 3B; PCDH7, protocadherin 7; HTRA1, high temperature requirement A serine peptidase 1; NTNG1, netrin G1; Th2, CD4^+^ T helper-2 cell; Th17, CD4^+^ T helper-17 cell; IFN-γ, interferon-γ; *cognate antigen for anti-NELL1, anti-Sema3B, anti-PCDH7, anti-serine protease HTRA1, and anti-NTNG1 autoantibodies respectively. **(A)**: 1. In Heymann nephritis, anti-Fx1A antibodies bind primarily to megalin on podocytes and activate the classical complement pathway (via the IgG2b subclass). 2. Anti-RAP antibodies are also detected and may represent epitope spreading due to the association between megalin and RAP, which assists the transport of megalin from ER to cell surface. 3. Complement activation may be potentially exacerbated by binding of anti-Fx1A antibodies to complement regulatory proteins Crry and CD59. 4. Sublethal C5b-9 injury causes intracellular calcium influx, activating cPLA2, which hydrolyzes membrane phospholipids of the podocyte, ER and nuclear envelope. This causes ER stress, generation of ROS, disruption of the slit diaphragm protein nephrin, and release of AA with subsequent COX-mediated generation of prostanoids (PGE2, TxA2) that increase glomerular filtration pressure, exacerbating proteinuria. Glomerular IgG and complement deposition attract glomerular infiltrates of CD8^+^ T cells, Th1 cells, and macrophages, potentially *via* the IgG Fc receptor and anaphylatoxins C3a/C5a. 5. Autoantibody production in Heymann nephritis is at least in part dependent on Qa-1-expressing Tfh cells, which are inhibited by CD8^+^ Tregs. **(B)**: 1. In primary membranous nephropathy (PMN), autoantibodies are predominantly directed against PLA2R, and 2. less commonly against THSD7A, 3. NELL1, PCDH7, serine protease HTRA1 and NTNG1, except in pediatric PMN where Sema3B is the most common podocyte antigen targeted by autoantibodies. 4. Increased B cells and Tfh cells are observed, which interact in the germinal center of lymph nodes to induce differentiation of B cells into high-affinity antibody-producing plasma cells. Autoantibodies in PMN are primarily of the IgG4 subclass, potentially due to Th2-mediated IL-4 production. Loss of tolerance to podocyte antigens such as PLA2R may be due to loss of thymus-derived podocyte antigen-specific Tregs. 5. Glycosylated anti-PLA2R IgG4 bind the MBL/MASP-1/2 complex to activate the lectin complement pathway. 6. This causes activation of C3aR and C5aR as well as deposition of C5b-9, which activates cathepsin L- and an aspartic protease-mediated proteolysis of the actin cytoskeleton protein synaptopodin and slit diaphragm protein NEPH1 respectively. Anti-PLA2R antibody-containing serum is also associated with reduced FH, impaired autophagy and reduced adhesion to the GBM, though it is unclear whether these effects are mediated *via* complement activation. 7. Impairment in autophagy results in internalization of nephrin and the actin cytoskeleton protein α-actinin-4. 8. Reduced FH leads to intracellular fumarate accumulation, which is associated with increased generation of ROS, reduced slit diaphragm protein ZO-1 and reduced transcription of WT-1, which normally activates the expression of podocyte proteins nephrin and podocalyxin. The mechanisms of podocyte injury of other autoantibodies associated with PMN remain incompletely understood.

AHN is characterized by anti-Fx1A autoantibodies without circulating IgG subclass predominance but induce proteinuria *via* complement-fixing rat IgG2b (equivalent to human IgG1) (**Figure 1A.1**) (57–59). The main pathogenic epitope on megalin at its N-terminal domain is glycosylation- and conformation-dependent though epitope spreading occurs, similar to PMN, whereby the immune response to a disease antigen may extend to non-cross-reactive peptide sequences on the same antigen (intramolecular spreading) or adjacent antigens (intermolecular spreading) over time (60, 61). Accordingly, anti-RAP autoantibodies likely represent intermolecular epitope spreading due to the association of RAP with megalin (**Figure 1A.2**).

### Passive administration of anti-PLA2R or anti-THSD7A antibodies in mice

For the passive administration of anti-PLA2R antibodies, transgenic mice expressing murine PLA2R were required due to a lack of PLA2R expression normally on murine podocytes (24). Murine PLA2R has 72% sequence homology with human PLA2R and adoptive transfer of human anti-PLA2R autoantibodies could not bind murine PLA2R. Instead disease induction required transfer of rabbit anti-PLA2R antibodies (24). Therefore, the pathogenicity of human anti-PLA2R antibodies remains to be definitively proven *in vivo*.

In contrast, murine podocytes normally express THSD7A. Interestingly, human sera containing anti-THSD7A antibodies but not purified human anti-THSD7A antibodies activated the lectin complement pathway on murine podocytes and caused oxidative stress, disruption of nephrin, and rearrangement of the podocyte actin cytoskeleton (25–27). In comparison, rabbit anti-THSD7A antibodies (with or without sera) induced significant proteinuria in the absence of complement activation (25, 27). These discrepant results could be related to lower amounts of injected purified human antibody, alteration of autoantibodies when purified using acid-elution or the presence of other proteins within human sera required for complement activation. Furthermore, it is unclear whether generation of rabbit anti-PLA2R and anti-THSD7A antibodies are predominantly of IgG4 subclass (like their human counterparts) and therefore whether disease pathophysiology caused by passive administration of these antibodies is homologous to human PMN (24, 27).

### Transgenic mice expressing human PLA2R specifically on podocytes

Full length human PLA2R was expressed on podocytes of these mice and anti-human PLA2R antibodies recognized the cysteine-rich domain as well as C-type lectin domains (CTLD) 1, 7 and 8, which are the same epitopes recognized by anti-PLA2R antibodies in patients with PMN (28, 62–64). Therefore, this represents the ideal model for confirming the pathogenicity of human anti-PLA2R autoantibodies.

## Antigens and autoantibodies in human membranous nephropathy

While there are many similarities between Heymann nephritis and PMN, they are not homologous primarily due to differences in the disease antigen. Megalin (called low-density lipoprotein receptor-related protein 2 (LRP2) in humans) is 77% homologous between rodents and humans, though human tissue expression is predominantly on proximal tubular brush border and glomerular staining is weak (56, 65). Accordingly, anti-LRP2 antibodies in humans cause ABBA (anti brush border antibody) disease, characterized by tubular immune deposits with segmental glomerular subepithelial deposits, subnephrotic proteinuria, and kidney failure (66).

Unlike Heymann nephritis, IgG4 is the predominant IgG subclass in most patients with PMN though IgG1 is predominantly found in neural epidermal growth factor-like 1 protein (NELL1)- and semaphorin 3B (Sema3B)-associated PMN without significant activation of the classical complement pathway based on rare C1q deposition (40, 41).

### PLA2R

PLA2R is the primary disease antigen in 70% of PMN and while the pathogenicity of anti-PLA2R autoantibodies is highly likely due to the strong correlation of antibody titres with disease activity, *in vitro* evidence of podocyte injury caused by anti-PLA2R sera and lectin pathway activation by anti-PLA2R IgG4, definitive *in vivo* evidence of pathogenicity is yet to be confirmed (**Figure 1B.1**) (3, 67–70). The major epitope targeted by anti-PLA2R antibodies is located at the 31-mers peptide sequence of the cysteine-rich domain of PLA2R (specifically the N-terminal linear stretch VIQSES and C-terminal stretch SVLTLENC regions) with additional antibody epitopes identified at CTLD1, CTLD7 and CTLD8 (62, 64, 71). Epitope spreading from the cysteine-rich domain to the CTLD1 and/or 7 domains occurs in 25-66% of patients over the course of 3 months to 2 years (72, 73). However, data are inconsistent regarding whether epitope spreading is associated with treatment resistance (64, 72, 73).

Genome-wide association studies have greatly enhanced our understanding of how self-antigens such as PLA2R trigger an autoimmune response. The risk of PMN was substantially increased in individuals with both single nucleotide polymorphisms (SNP) in class II human leukocyte antigen (HLA-DQA1*0501 in Europeans, -DRB1*1501 in East Asians, and -DRB1*0301 in both ethnicities) on antigen-presenting cells (APC) and SNPs in the PLA2R locus (rs4664308 in Europeans and rs17831251 in both Europeans and East Asians) (74, 75). These non-coding region SNPs were either associated with other SNPs within the PLA2R coding region or an enhancer element resulting in an altered amino acid sequence or increased tissue expression respectively (75). This may enhance antigen presentation by APCs expressing risk HLA variants to their cognate CD4^+^ T cell, thereby initiating the process of autoimmunity. *In silico* analysis predicted HLA-DRB1*1501 and HLA-DRB1*0301 preferentially bound to PLA2R peptides in the CTLD1, CTLD7 and between CTLD4 and CTLD5 regions compared to the 31-mers peptide, though experimental validation is required (76).

In recurrent PLA2R-associated MN after kidney transplantation, detectable anti-PLA2R autoantibody titres pre-transplantation and steroid-free immunosuppression are known risk factors (16, 77–79). Positive glomerular staining for PLA2R in these cases also suggest the same antigen is implicated in recurrent MN (78). However, the HLA risk alleles (HLA-DQA1*0501, -DRB1*1501, and -DRB1*0301) associated with PMN were not associated with recurrent MN but rather two non-coding HLA-D SNPs (rs9271550 and rs9271705) and three PLA2R SNPs (rs3828323, rs17830558, and rs3749117)) when present on the donor but not the recipient (80). Another study found an association between recurrent MN and recipient HLA-A3 (81). Discrepancies between studies may be due to the relatively small sample sizes and different diagnostic criteria for recurrent MN. Overall, the pathogenesis of recurrent MN remains poorly understood and further studies are required.

### THSD7A

THSD7A was the second podocyte antigen discovered in PMN, implicated in 10% of PLA2R-negative PMN (**Figure 1B.2**) (4). THSD7A and PLA2R are structurally similar as are their associated autoantibodies, which are predominantly of IgG4 subclass, activate complement with glomerular C5b-9 staining, bind initially to the immunodominant epitope on the N-terminal domain of their target antigen exclusively in non-reducing conditions, and are associated with epitope-spreading (4, 25, 63, 82). Contrary to the prevailing paradigm of a single podocyte antigen being targeted in PMN, dual anti-PLA2R and anti-THSD7A antibodies and staining on biopsy has been reported in 1% of cases (83).

### Other antigens implicated in primary membranous nephropathy

Laser microdissection and mass spectrometry on kidney biopsies of PLA2R-negative PMN has led to the discovery of many more disease antigens such as NELL1, Sema3B, protocadherin 7 (PCDH7), high temperature requirement A serine peptidase 1 (HTRA1), and netrin G1 (NTNG1) (**Figure 1B.3**). These antigens account for less than 10% of PMN and their associated autoantibodies vary in their predominant IgG subclass, ability to activate complement, and the association of their pathogenic epitope with disulfide bonds based on whether autoantibodies were detected under reducing or non-reducing conditions (**Table 1**) (40, 41, 43–50). Anti-Sema3B autoantibodies correlated with disease activity in a case report of severe pediatric PMN, suggestive of pathogenicity (84). However, the pathogenicity of other autoantibodies remains to be determined and the mechanism by which these self-antigens trigger an autoimmune response remain unclear. Furthermore, it remains unclear how some of these antigens such as NELL1 and PCDH7 display weak or no staining in normal glomeruli but are implicated in PMN, whereby the subepithelial localization of deposits on electron microscopy in NELL1- and PCDH7-associated MN strongly suggest shedding from podocytes rather than mesangial or endothelial cells. NELL1 and PCDH7 are known to be secretory proteins and highly glycosylated proteins respectively (85), which may impact their expression under healthy conditions. However, further research on the expression and localization of these novel antigens in normal podocytes are required. Overall, it is apparent that PMN is not a single disease entity but rather a group of diseases characterized by autoantibodies against different antigens causing kidney-limited autoimmune disease.

### Antigens implicated in secondary and neonatal membranous nephropathy

MN podocyte antigens exostosin 1/eoxstosin 2 (EXT1/EXT2), neural cell adhesion molecule 1 (NCAM1), and transforming growth factor-β receptor 3 (TGFBR3) are associated with other autoimmune diseases, especially systematic lupus erythematosus (SLE) (46–48). In particular, EXT1/EXT2-associated membranous lupus nephritis confers a better prognosis than EXT1/EXT2-negative membranous lupus nephritis (86). This may be due to EXT1/EXT2-related glycosylation of heparan sulfate, which protects the GBM against immune injury. Contactin 1 (CNTN1) is associated with chronic inflammatory demyelinating polyneuropathy-related MN and protocadherin FAT1 (FAT1) is associated with allogeneic hematopoietic stem cell transplant-related MN (49, 50).

In contrast, while MN secondary to infections such as hepatitis B have been well described and hypothesized to be due to molecular mimicry, the associated podocyte antigen remains unclear (87). Evidence of molecular mimicry due to malignancy is more convincing for THSD7A- and NELL1-associated MN. THSD7A was found in tumor and follicular dendritic cells of tumor-infiltrated lymph nodes in separate cases of gallbladder adeno-neuroendocrine carcinoma and endometrial carcinoma (88, 89) Up to 33% of NELL1-associated MN were secondary to malignancy with NELL1 tumor expression demonstrated in 2 cases (invasive ductal carcinoma of the breast and follicular lymphoma) (90). Lastly, neutral endopeptidase (NEP/CD10) is a podocyte antigen implicated in neonatal MN, which is caused by placental transfer of anti-NEP antibodies to the fetus from NEP-deficient mothers (truncating mutations in the *MME* gene) who undergo alloimmunization during the pregnancy or from previous miscarriages (51, 91).

### Other antigens reported in primary membranous nephropathy

IgG4 against aldose reductase (AR) and superoxide dismutase 2 (SOD2) have also been described in multiple PMN cohorts (92, 93). However, these antigens are not expressed on the surface of normal podocytes, antibody titres do not correlate with disease activity, and both AR and SOD2 are involved in oxidative stress, a known downstream effect of podocyte injury (92, 93). Accordingly, *in vitro* and *in vivo* evidence demonstrated podocyte membrane expression of SOD2 increasing with intracellular oxidation and after passive transfer of anti-THSD7A IgG respectively (27, 92). These data suggest antibodies targeted AR and SOD2 are a secondary phenomenon to autoantibody-mediated podocyte injury exposing neoantigens rather than autoantibodies driving PMN pathogenesis. More recently, autoantibodies against Formin-like 1 (FMNL1) were detected in patients with PMN as well as other forms of glomerular disease (IgA nephropathy and focal segmental glomerulosclerosis), which recognize FMNL1 expression on macrophages (94). Whether FMNL1 positive macrophages contribute to the pathogenesis of PMN (ie. M1 macrophages) or highlight a reparative role of macrophages (ie. M2 macrophages) in PMN requires further study.

## T and B cell immunity in animal models of membranous nephropathy

### Heymann nephritis

There is a clear T cell role in Heymann nephritis with CD4^+^ T cell depletion abolishing IgG and C3 deposition as well as proteinuria, demonstrating the essential role of CD4^+^ T cell-B cell interaction in autoantibody formation (95). In contrast, CD8^+^ T cell depletion reduced proteinuria with intact glomerular IgG and C3 deposition in AHN (59, 96), and reduced proteinuria in the autologous phase (week 2-4) in PHN but not during the heterologous phase (week 1) where infused anti-Fx1A antibodies cause complement-mediated podocyte injury (97). Therefore, the small CD8^+^ T cell infiltrate seen in Heymann nephritis likely occurs downstream of podocyte antibody deposition and proteinuria.

Development of anti-Fx1A autoantibodies in AHN suggest loss of immune tolerance, likely due to escape from thymic deletion by megalin-specific effector T cells and/or impaired induction of megalin-specific Foxp3^+^ CD4^+^ Tregs, though overall levels of Foxp3^+^ CD4^+^ Tregs are unchanged in AHN (98). In contrast, evidence of regulation that dampen autoimmunity in AHN were found to be mediated by thymus-derived CD8^+^ T cells, which reduced autoantibody production and proteinuria upon adoptive transfer (99, 100). In particular, CD8^+^ Tregs reduced anti-Fx1A antibody production in AHN by targeting CD4^+^ T follicular helper (Tfh) cells expressing the non-classical MHC class I molecule Qa-1 (**Figure 1A.5**). In PMN, CD8^+^ Tregs have yet to be described while CD4^+^ Tregs were reduced in patients with active disease prior to receiving immunosuppression (101).

### Transgenic mice expressing human PLA2R specifically on podocytes

Tomas et al. demonstrated that human PLA2R was not expressed in the thymus of their transgenic mice, explaining the spontaneous formation of anti-human PLA2R antibodies is likely due to a lack of central tolerance (28). In contrast, another study described transgenic knock-in mice with ubiquitous expression of human PLA2R that did not develop a PMN phenotype, likely due to establishment of immune tolerance to human PLA2R, though expression of human PLA2R in the thymus of these mice was not reported (102). Lastly, in the study by Tomas et al, a PMN phenotype did not develop in recombination activating gene (RAG) 2 knock-out mice expressing human PLA2R specifically on podocytes, indicating the requirement of mature T and B cells for the formation of anti-human PLA2R autoantibodies (28).

## T and B cell and innate immunity in human membranous nephropathy

Study of T cell subsets in PMN have revealed an increased CD4^+^:CD8^+^ T cell ratio (103, 104). Further analysis of CD4^+^ T cells in PMN showed increased Th2 cells without change in Th1 cells, increased Tfh cells, and lower CD4^+^ Tregs, which were associated with increased IgG4 production, increased plasma cells and a loss of immune tolerance respectively (**Figure 1B.4**) (101, 103, 105–108). Our understanding of PMN disease antigens and class II HLA linkage suggest that HLA-driven effector T cells and/or the loss of Tregs likely play a role in loss of immune tolerance, similar to Goodpasture disease, another autoimmune kidney disease caused by autoantibodies directed against a glomerular antigen (109). Indeed, reduced CD4^+^ Treg number and/or function have been described in various autoimmune diseases, demonstrating its critical role in maintaining immune tolerance (110–113). Accordingly, enhanced ability of Tregs to suppress the anti-PLA2R response in PMN has also been hypothesized to be the mechanism underlying the spontaneous remission observed in a third of patients, however data supporting this theory are lacking (114).

More recently, increased Th17 cells have been demonstrated to correlate with disease activity, relapse and thrombotic complications in PMN (115, 116), and IL-17 signaling was differentially expressed in single-cell RNA sequencing of kidney biopsy specimens in patients with PLA2R-associated PMN (117). Whether Th17 activation is triggered by glomerular antibody deposition, proteinuria or tissue injury requires further study. Single-cell RNA sequencing of kidney biopsy specimens in PMN also identified tumor necrosis factor (TNF) signaling and NOD-like receptor signaling in glomerular endothelial cells, pericytes and tubular cells compared to healthy controls, suggesting a role of innate immunity (117). In comparison, bulk RNA sequencing and microarray expression profiles of the glomerular compartment of kidney biopsies revealed a PMN-specific signature significantly enriched in NF-kB1 targets compared to other glomerulopathies, which is consistent with a GWAS finding of a variant at the NF-kB1 locus conferring an increased risk of PMN (75, 118). Regarding the significance of TNF signaling in PMN, circulating TNF receptors correlated with proteinuria and tubular TNF receptor expression but not anti-PLA2R autoantibody titres, which may reflect a downstream effect of heavy proteinuria on stimulating tubular cell chemokine production and infiltration of TNF-α-producing macrophages and lymphocytes (119, 120). Indeed, TNF inhibition reduced kidney inflammatory infiltrates but not proteinuria or immune complex deposition in BSA-induced murine MN, highlighting the need to validate the findings of transcriptomic profiling with *in vivo* studies to improve our understanding of disease pathophysiology (121).

Analysis of B cell populations in PMN have demonstrated an increase in naive B cells and reduced memory B cells, which could be secondary to Tfh-mediated differentiation of B cells into autoantibody-producing plasma cells or B cell infiltration into the kidney (104, 122). B cell depletion with rituximab was associated with increased CD4^+^ Tregs, potentially due to B cell expression of interferon-γ, which suppressed CD4^+^ Tregs in animal studies (**Figure 1B.4**) (104, 123, 124). Regulatory B cells (Bregs) have been implicated in the suppression of B-cell-mediated autoimmunity through IL-10 production. Breg levels in PMN have varied between studies due to differences in surface markers assessed (101, 125, 126), though overall higher Bregs correlated with better treatment responses (101, 125).

Despite the rapid discovery of antigens targeted by autoantibodies in PMN, identification of antigen-specific T and B cells has yet to be described. T cell receptor (TCR) gene sequencing in patients with PMN revealed lower diversity of the VDJ cassette combination compared to healthy controls, which may represent expansion of antigen-specific T cells (127). The same group analyzed the B cell receptor (BCR) sequences in patients with PMN and found increased somatic hypermutation and length distribution of the third complementarity-determining region of the heavy chain (CDR-H3) of all immunoglobulin isotypes compared to healthy controls, indicative of overall increased B cell activation in PMN (128). Future studies linking TCR and BCR sequences with pathogenic epitopes of PLA2R (or other antigens implicated in MN) may assist in identifying autoreactive T and B cells.

## The complement system in animal models of membranous nephropathy

### Heymann nephritis

**Figure 2:**
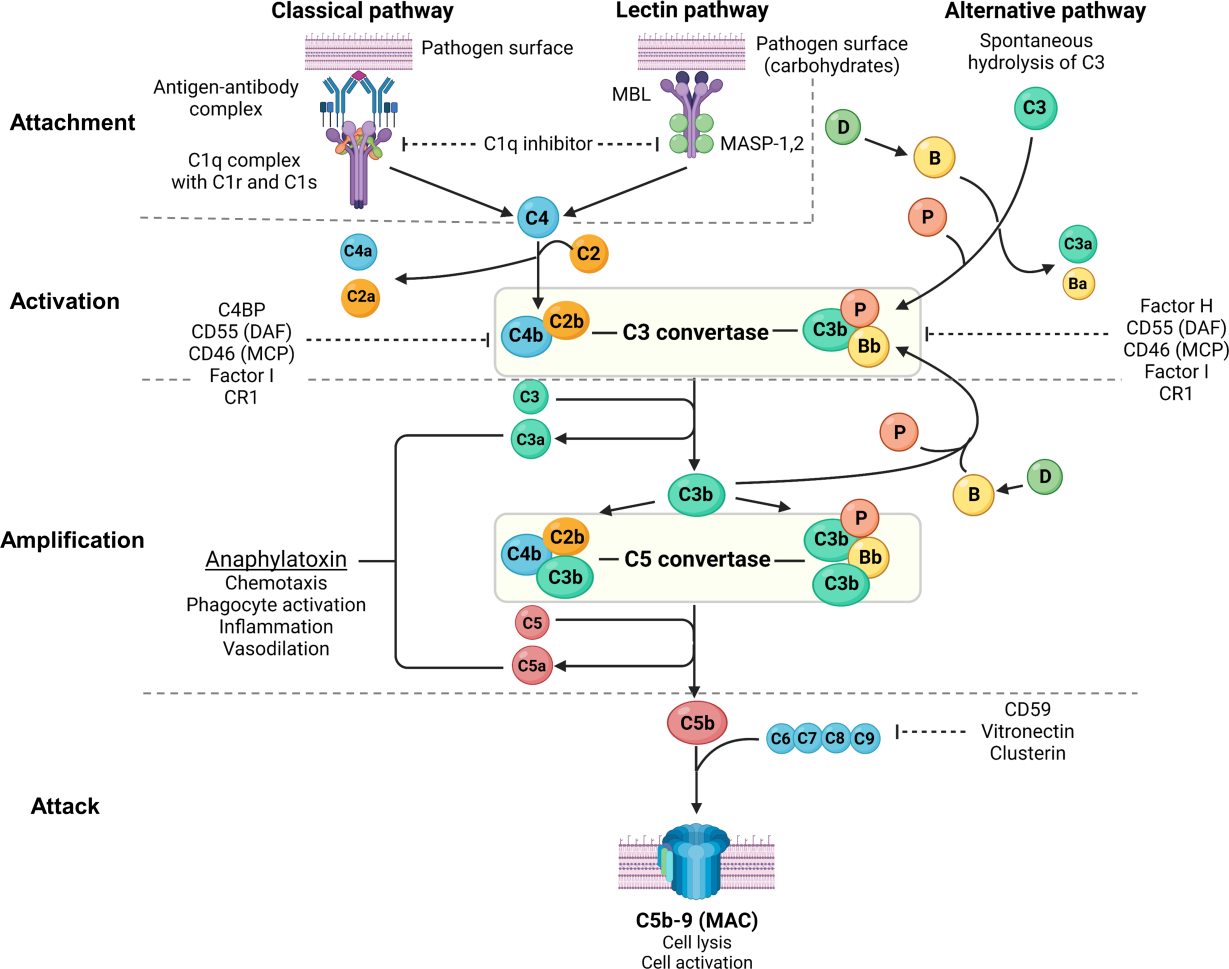
Overview of the complement system. Created with BioRender.com. MBL, mannan-binding lectin; MASP, mannan-binding lectin serine protease; C4BP, C4b-binding protein; DAF, decay-accelerating factor; MCP, membrane cofactor protein; CR1, complement receptor 1; MAC, membrane **attack** complex; C3aR, C3a receptor; C5aR, C5a receptor; PMN, primary membranous nephropathy; PLA2R, M-type phospholipase A2 receptor 1; THSD7A, thrombospondin type-1 domain-containing protein 7A. The complement system consists of 3 pathways: classical pathway, lectin pathway and alternative pathway, which can be conceptually understood using 4A’s: attachment, activation, amplification, and attack (steps of complement activation in bold text below). In the classical pathway, complement C1q **attaches** to the Fc receptor of antigen-bound antibodies (IgG1, IgG3 and IgM in humans, and IgG2a and IgG2b in rodents). The lectin pathway is similar to the classical pathway where lectins (such as ficolins, MBLs or collectins) **attach** to carbohydrates on pathogens, activating MASP-1 and MASP-2 (analogous to C1r and C1s of the classical pathway), which cleave C4 and C2 to form C3 convertase (C4b2b). In the alternative pathway, small amounts of C3 are spontaneously **activated** (“C3 tickover”) due to a labile thioester bond, forming a C3b fragment that binds to factor B (B), which in turn is cleaved by factor D (D) to form the alternative pathway C3 convertase (C3bBb) that is stabilised by properdin (P), forming C3bBbP. Regardless of the pathway of activation, C3 convertase cleaves C3 into C3a and C3b, which **amplifies** alternative pathway activation whereby further factor B binds to C3b forming more C3 convertase. Apart from amplifying C3 convertase formation, C3b also acts as an opsonin and binds to C3 convertase to form C5 convertase (C4b2b3b and C3bBbC3bP), which cleaves C5 into C5a and C5b. C3a and C5a act as potent anaphylatoxins *via* their respective receptors (C3aR and C5aR). Complement **attack** is initiated by C5b sequentially binding C6, C7, C8, and C9 to form the MAC (C5b-9), which causes transmembrane pores that cause osmotic cell lysis or sublethal cell injury, activating internal cellular processes. The complement system is tightly regulated by proteins present in plasma (fluid-phase) and on cell surfaces including the podocyte (solid-phase). Fluid-phase regulators include C1q inhibitor (binds C1r/C1s and MASP-1/MASP-2 to prevent classical and lectin pathway activation), C4BP (binds C4b to prevent formation of the classical/lectin pathway C3 convertase), factor H (binds C3b to prevent formation of the alternative pathway C3 convertase), factor I (cofactor for factor H and MCP, which both inhibit C3 convertase formation), vitronectin (binds the C5b-7 complex to prevent MAC formation) and clusterin (binds C7, C8, C9 to prevent MAC formation). Solid-phase regulators include DAF (dissociates C2b from C4b and Bb from C3b to inactivate C3 convertase), MCP (binds C3b and C4b to prevent C3 convertase formation), CR1 (binds C3b and C4b to prevent C3 convertase formation) and CD59 (binds C8 and C9 to prevent MAC formation).

While megalin alone could recapitulate AHN, immunization with Fx1A caused more severe proteinuria suggesting additional antigens targeted by anti-Fx1A autoantibodies contributed to podocyte injury (129). Such antigens include solid-phase complement regulatory proteins such as Crry (murine equivalent of complement receptor 1 (CR1)) and CD59 (**Figure 1A.3**, **Figure 2**). Indeed, Crry-deficient Fx1A could not induce AHN without administration of anti-Crry antibodies (130). Lower glomerular CR1 and CD59 are reported in PMN though without evidence of associated autoantibodies and may simply represent podocyte loss (131, 132). Overall, these data suggest complement activation plays an important role in mediating podocyte injury after antibody deposition in Heymann nephritis.

The crucial role of complement-mediated glomerular injury in Heymann nephritis was demonstrated by the prevention of proteinuria in PHN with complement depletion using cobra venom factor (133). In AHN, proteinuria was dependent on glomerular deposition of rat complement-fixing IgG2b suggesting classical pathway activation (57, 58), and increased podocyte expression of complement factor H (CFH) in PHN also suggests alternative pathway activation (134). More recently, classical and alternative pathway but not lectin pathway activation was confirmed in PHN with associated increases in plasma C3a and glomerular expression of C3a receptor (C3aR). Accordingly, C3aR antagonism ameliorated disease by inhibiting the downregulation of synaptopodin (actin-associated protein in podocytes) and Bcl2 (apoptosis inhibition gene), and the upregulation of β-catenin on podocytes, which was recapitulated in human podocyte cell lines exposed to PMN sera (135). Interestingly, C3aR antagonism did not alter inflammatory cell infiltration (including CD4^+^ T cells, CD8^+^ T cells, and macrophages) in the kidney of PHN rats despite C3a, an anaphylatoxin, being a potent chemoattractant (135).

Glomerular deposition of terminal complement component C5b-9 has been demonstrated in both Heymann nephritis and PMN (70). PVG/c rats with or without C6 deficiency induced with PHN or AHN demonstrated no differences in proteinuria, though C6 depletion using goat anti-rat C6 IgG in Sprague-Dawley rats with PHN significantly reduced proteinuria (97, 136, 137). Nevertheless, C5b-9 has been shown to cause sublethal podocyte injury, leading to activation of cytosolic phospholipase A2, oxidative stress, lipid peroxidation, DNA damage, and disruption of the podocyte slit diaphragm (**Figure 1A.4**) (138).

## The complement system and other mechanisms of Glomerular injury in human membranous nephropathy

Despite glomerular IgG and complement deposition being well-described in PMN, this finding long appeared to be inconsistent with the predominance of non-complement fixing IgG4. While there is a predominance of complement-fixing IgG1 deposition in early stages of PMN (139, 140), the lack of glomerular C1q but almost universal C4d deposition suggested activation of the lectin pathway rather than the classical pathway (**Table 2**) (141). Indeed, glomerular deposition of mannan-binding lectin (MBL) co-localized with anti-PLA2R IgG4 and correlated with disease activity (142, 143). Recently, Haddad et al. provided important insights into the mechanism of lectin pathway activation in PMN by showing glycosylation patterns on anti-PLA2R IgG4 allow binding of MBL (**Figure 1B.5**). Such glycosylation patterns were not demonstrated in IgG4 of PLA2R-negative PMN, secondary MN or other glomerular diseases, demonstrating distinct autoimmune responses against different antigens implicated in PMN. Furthermore, anti-PLA2R IgG4 but not IgG4-depleted sera from patients with PLA2R-associated PMN was essential for the proteolysis of two podocyte proteins synaptopodin and NEPH1 *via* C3aR and C5aR activation on podocytes (**Figure 1B.6**), which could be prevented by inhibiting MBL-associated serine protease (MASP) or using C6-depleted sera (70). Increased serum C3a and glomerular expression of C3aR have also been demonstrated in PMN patients, which correlated with disease activity and was associated with increased podocyte expression of PLA2R and reduced expression of synaptopodin (135).

**Table 2:** Complement system involvement in Heymann nephritis and membranous nephropathy.

Component of the complement pathway	Change in membranous nephropathy	Comments
Classical pathway		
C1q	HN: ↑ PMN: ↔ SMN: ↑/↔	Glomerular deposition of IgG2b in HN binds C1q. IgG4 (predominant IgG subclass in PLA2R-, THSD7A-, PCDH7-, HTRA1- and NTNG1-associated PMN) does not bind C1q. Predominant IgG1 (NELL-1- and Sema3B-associated PMN) not associated with C1q deposition. IgG1 (predominant IgG subclass in EXT1/EXT2- and NCAM-1-associated MN) binds C1q. IgG4 (predominant IgG subclass in CNTN1- and FAT1-associated MN) does not bind C1q.
Lectin pathway		
MBL	HN: ↔ PMN: ↑ SMN:?	Glomerular MBL staining negative in HN. Glycosylated anti-PLA2R IgG4 allows MBL binding. MBL deposition also demonstrated in THSD7A-associated PMN.
Alternative pathway		
Factor B/Properdin	HN: ↑ PMN: ↑ SMN:?	Increased glomerular deposition of factor B in HN. Factor B and properdin deposition in PMN patients with MBL deficiency. Mechanism of activation unclear.
Common pathway		
C3	HN: ↑ PMN: ↑ SMN: ↑	C3 deposition is a hallmark of HN and MN (primary and secondary). Increased serum C3a and glomerular expression of C3aR demonstrated in both HN and PMN.
C5b-9	HN: ↑ PMN: ↑ SMN:?	Glomerular deposition of C5b-9 in HN. C5b-9 deposition demonstrated in PLA2R- and THSD7A-associated PMN.
Complement regulatory proteins		
Fluid-phase	HN: ↑ PMN: ↑ SMN:?	Increased factor H reflecting alternative pathway activation. Increased factor H reflecting alternative pathway activation. Increased vitronectin and clusterin reflecting C5b-9 activation.
Solid-phase	HN: ↓ PMN: ↓ SMN:?	Anti-Fx1A antibodies bind Crry and CD59. Decreased CR1 and CD59, potentially reflecting podocyte loss.

"↑" = increase, "↔" = no change, "↓" = decrease, and "?" is unknown. HN, Heymann nephritis; PMN, primary membranous nephropathy; SMN, secondary membranous nephropathy; NA, not applicable; PLA2R, M-type phospholipase A2 receptor 1; THSD7A, thrombospondin type-1 domain-containing protein 7A; NELL-1, Neural epidermal growth factor-like 1 protein; Sema3B, Semaphorin 3B; CNS, central nervous system; PCDH7, Protocadherin 7; NTNG1, netrin G1; SLE, systemic lupus erythematosus; HTRA1, high temperature requirement A serine peptidase 1; EXT1/EXT2, exostosin 1/exostosin 2; NCAM-1, neural cell adhesion molecule 1; CNTN1, contactin 1; FAT1, protocadherin FAT1; MBL, mannan-binding lectin; C3aR, complement 3a receptor; CR1, complement receptor 1.

Despite growing evidence of lectin pathway activation in PMN, PLA2R-associated PMN has also been described in patients with MBL deficiency in whom disease activity was mediated by alternative pathway activation (144). However, the mechanism of alternative pathway activation in PMN without initial activation of the lectin pathway remains unclear though one could postulate the role of traditional activators of the alternative pathway such as infection, which increase C3a levels and subsequent overexpression of PLA2R on podocytes that predisposes to autoimmunity. Anti-CFH autoantibodies are an unlikely mechanism of alternative pathway activation, detected in only 3% of patients with PMN (145). CFH also binds to human glomeruli *via* interaction with heparan sulfate, which is downregulated in PMN, AHN and PHN though whether this results in alternative pathway activation remains unproven (146–149).

### Other mechanisms of glomerular injury in primary membranous nephropathy

Impaired podocyte autophagy, fumarate accumulation in podocytes causing oxidative stress, and disruption of podocyte adhesion to collagen IV in the GBM have also been implicated in glomerular injury in PMN. Impairment of podocyte autophagy in PMN causes internalization of nephrin and α-actinin-4 (podocyte actin cytoskeleton protein), which in PLA2R-associated PMN may be mediated by activation of the mTOR/ULK1^ser757^ signaling pathway (**Figure 1B.7**) (150–153). Fumarate hydratase, which co-localizes with podocalyxin on podocytes, is reduced in PLA2R-associated PMN in an oxidative stress- and C5b-9-dependent manner (**Figure 1B.8**) (154). Inhibition of fumarate hydratase using small interfering RNA results in fumarate accumulation within podocytes causing oxidative stress and reduction in zonula occludens-1 (podocyte slit diaphragm protein) and Wilm’s tumor 1 (podocyte transcription factor) (154, 155). Lastly, *in vitro* evidence suggests human sera containing anti-PLA2R antibodies impair podocyte adhesion to collagen IV on the GBM though the exact mechanism remains unclear (156).

## Novel therapeutics: Potential targets and challenges

### Targeting autoantibody production by B cells and plasma cells

**Figure 3:**
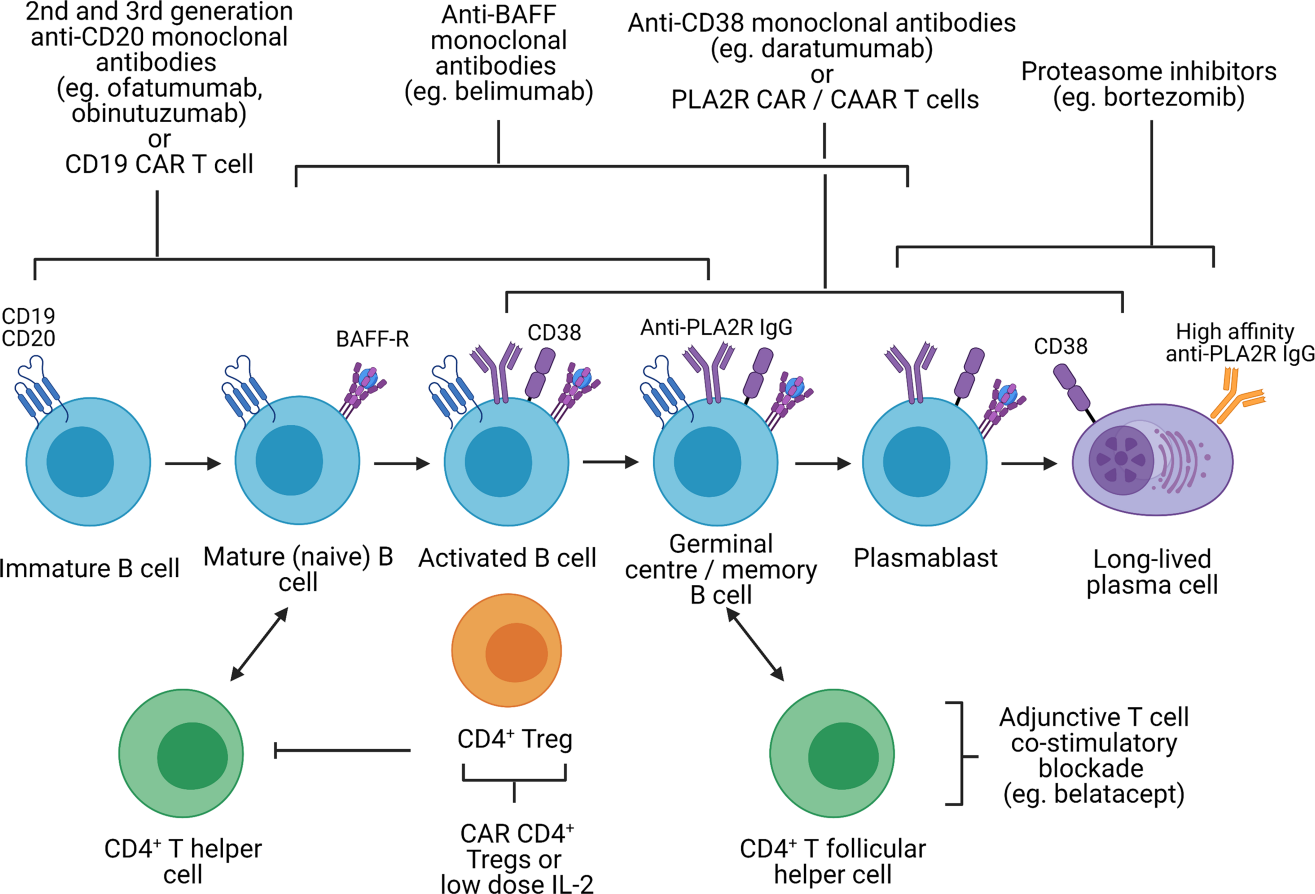
Potential immunological targets for novel therapeutics in primary membranous nephropathy. Created with BioRender.com. BAFF-R, B-cell activating factor receptor; CAR, chimeric antigen receptor; CAAR, chimeric autoantibody receptor; PLA2R, M-type phospholipase A2 receptor 1; Treg, regulatory T cell, In primary membranous nephropathy, autoreactive B cells produce autoantibodies targeting podocyte antigens (represented by PLA2R in this figure but also include thrombospondin type-1 domain-containing protein 7A, neural epidermal growth factor-like 1 protein, semaphorin 3B, protocadherin 7, high temperature requirement A serine peptidase 1, and netrin G1). These autoreactive B cells originate from the bone marrow, mature from immature B cells to mature B cells, become activated upon interaction with CD4^+^ T helper cells (via cytokines and CD40 ligation) and undergo isotype switching. Activated B cells subsequently migrate to the germinal center of secondary lymphoid organs where they interact with CD4^+^ T follicular helper cells, resulting in B cell proliferation and somatic hypermutation, ultimately differentiating into plasmablast and long-lived plasma cells which secrete high-affinity autoantibodies. Immature, mature, activated and germinal center B cells express CD19 and CD20, which can be targeted by 2^nd^ and 3^rd^ generation anti-CD20 monoclonal antibodies (eg. ofatumumab and obinutuzumab), which may be superior to rituximab, or CD19 CAR T cells. However, in cases of primary membranous nephropathy resistant to 1^st^ generation anti-CD20 monoclonal antibodies (eg. rituximab) that respond to 2^nd^ or 3^rd^ generation anti-CD20 monoclonal antibodies, this may be due to changes in the CD20 antigen that may be restricted to autoreactive B cells. CD19 and CD20 are also not expressed on plasmablasts or long-lived plasma cells which continue to secrete autoantibodies despite B cell depletion. Monoclonal antibodies targeting BAFF (eg. belimumab), preventing binding to the BAFF receptor, target both B cells and plasmablasts but not long-lived plasma cells. Proteasome inhibitors have been shown to be effective in depleting plasmablasts and plasma cells. Anti-CD38 monoclonal antibodies (eg. daratumumab) may also be effective against plasma cells, plasmablasts and B cells. However, proteasome inhibitors and anti-CD38 monoclonal antibodies are associated significant toxicity and short-duration therapy to deplete plasma cells followed by adjunctive therapy (eg. B cell depletion or T cell co-stimulatory blockade to prevent B cell activation) may be better tolerated. With the discovery of disease antigens underpinning primary membranous nephropathy, CAR T cells or CAAR T cells targeting autoantibodies (eg. anti-PLA2R) on autoreactive B cells, plasmablasts and plasma cells are conceivable. Lastly, harnessing the capacity of CD4^+^ regulatory T cells to induce immune tolerance at sites of inflammation independent of antigen specificity (infectious tolerance) is attractive. Such strategies may involve CAR regulatory T cells directed to the kidney (eg. targeting PLA2R on the podocyte) or low-dose IL-2, which preferentially expands regulatory T cells not effector T cells.

PMN appears to be different disease entities rather than a uniform disease but with a unified immunological phenotype of autoantibody formation against an antigen mostly expressed on podocytes. Therefore, targeting antibody generation through B-cells, plasma cells and Tfh cells is an attractive strategy (**Figure 3**). While rituximab, a chimeric anti-CD20 monoclonal antibody, is effective at depleting B cells, treatment response in PMN is variable (60-85% complete or partial remission at 24 months) (12, 15). This may be related to autoantibody production by CD20-negative plasma cells, changes in the CD20 antigen that may be restricted to autoreactive B cells, or the development of neutralizing antibodies against rituximab. Ofatumumab and obinutuzumab are humanized anti-CD20 monoclonal antibodies, which have successfully treated patients with PMN who developed serum sickness to rituximab or anti-rituximab antibodies (157, 158), and demonstrated superior B-cell depletion compared to rituximab respectively (159). The efficacy of binutuzumab and belimumab [humanized monoclonal antibody against B-cell activating factor (BAFF)] in lupus nephritis, another disease mediated in part by autoreactive B cells, and CD19 CAR T cells in treatment-refractory SLE are also promising alternative B cell-depleting therapies, which require further evaluation in PMN (160–163).

Treatment resistance to B-cell depletion may also be attributable to persistent memory plasma cells, which produce autoantibodies independent of T and B cell interactions. The proteasome inhibitor bortezomib effectively depletes plasma cells and has been effective in both treatment-resistant and treatment-naïve PMN (164, 165). Plasma cells express CD38 and CD138, which could be targeted by anti-CD38 monoclonal antibodies such as daratumumab. However, safety concerns of neurotoxicity with bortezomib and infection with both bortezomib and daratumumab may limit their use. An alternative approach could be a short course of plasma cell-depleting therapy followed by B cell depletion or T cell co-stimulatory blockade to inhibit the Tfh-mediated B cell differentiation into plasma cells. In an uncontrolled cohort study and a case series of kidney transplant recipients with antibody-mediated rejection, short-duration bortezomib (on average 4 doses of 1.3 mg/m^2^) followed by adjunctive therapy (including rituximab, plasmapheresis, or intravenous immunoglobulin) or T cell co-stimulatory blockade with belatacept respectively were effective (166, 167). However, controlled clinical trials are needed to confirm the efficacy of these treatment regimens.

### Targeting antigen-specific B and T cells

The discovery of the disease antigens driving PMN also raises the possibility of antigen-specific therapeutics such as CAR or CAAR T cell therapies against PLA2R-specific B cells. In pemphigus vulgaris, an antibody-mediated autoimmune blistering skin disease, CAAR T cells expressing the disease autoantigen desmoglein 3 was able to eliminate desmoglein 3-specific B cells in a mouse model even in the presence of circulating autoantibodies and without off-target toxicity (168). A similar approach is conceivable in PMN where B cell-mediated autoimmunity is driven by a single autoantigen in most cases.

### Establishing immune tolerance

Inducing immune tolerance without abrogating anti-infection and anti-tumor immunity is the ultimate therapeutic strategy. CAR CD4^+^ Tregs targeting organ-specific antigens suppressed autoimmunity in animal models of type 1 diabetes, multiple sclerosis and autoimmune colitis (169–171). In particular, CAR Tregs in the autoimmune colitis model were able to suppress disease activity despite being specific for an antigen distinct from the disease antigen (171). This demonstrates the phenomenon of infectious tolerance whereby CD4^+^ Tregs can confer suppressive activity to conventional T cells *via* membrane-bound transforming growth factor-β (TGF-β) (172, 173). PLA2R may represent an ideal tissue marker to direct CAR Tregs to the glomerulus in PMN. An alternative strategy may be selective expansion of Tregs using low-dose IL-2, which was effective in animal models of rheumatoid arthritis and autoimmune colitis (174). However, a mechanism of IL-2 delivery to the kidney remains unclear.

Two distinct regulatory T cell subsets also exist to maintain immune tolerance at the germinal center: CD8^+^ Tregs and T follicular regulatory (Tfr) cells. In a mouse model of multiple sclerosis, yeast libraries have been used to identify peptides that engage the CD8^+^ Treg TCR and vaccination of these peptides suppressed disease activity (175). Neuritin, a protein produced by Tfr cells to prevent differentiation of B cells into plasma cells has also been shown to suppress autoantibody production in Tfr-deficient mice (176). However, the importance of CD8^+^ Tregs and Tfr cells in PMN remains to be established and development of these promising though experimental therapies will require rigorous pre-clinical evaluation.

### Are current animal models suitable for preclinical testing of novel therapeutics?

Heymann nephritis has significantly contributed to our understanding of the disease mechanisms causing PMN but distinct differences in the underlying disease antigen, predominant autoantibody IgG subclass, and mechanism of complement activation and glomerular injury remain. Despite this, AHN remains a relevant and feasible model that recapitulates the unifying disease phenotype of PMN and therefore is suitable for testing novel therapeutics targeting autoantibody production. Activation of the alternative complement pathway is also a hallmark of both AHN/PHN and PMN with C3aR antagonism ameliorating disease in PHN (135), and an ongoing trial will evaluate the factor B inhibitor iptacopan (LNP023) compared to rituximab in PMN (NCT04154787).

Models of PMN induced by passive administration of anti-PLA2R and anti-THSD7A autoantibodies may better resemble the mechanism of glomerular injury in human PMN. However, PLA2R and THSD7A in these models were not humanized, reflecting the inability of transferred human anti-PLA2R autoantibodies or purified human anti-THSD7A to recapitulate disease. Furthermore, rabbit anti-THSD7A antibodies lack the ability to activate complement, a hallmark of THSD7A-associated PMN. These animal models also require the passive administration of antibodies and therefore do not reproduce the immunological mechanism of autoantibody production in PMN.

Most recently, transgenic knock-in mice expressing human PLA2R specifically on podocytes faithfully recapitulates human PMN phenotype, representing an important and exciting development that opens opportunities to further understand the disease mechanisms driving PMN and test novel antigen-specific therapeutics such as CAR or CAAR T cells. It remains unclear whether a similar approach can be replicated to model MN associated with non-PLA2R antigens, though developing transgenic mice with podocyte-specific expression of the numerous non-PLA2R MN disease antigens present a significant challenge as well as feasibility issues. An alternative approach would be to immunize susceptible murine strains with a specific PMN-associated antigen and adjuvant. However, while expression of Sema3B and serine protease HTRA1 have been demonstrated in murine podocytes, PCDH7 is not detectable in rat kidneys and it is unclear whether murine podocytes express NELL1 or NTNG1 (42, 177–179).

## Conclusion

Our understanding of PMN is rapidly evolving with the promise of targeted therapeutics now emerging. Integrating technologies such as mapping T and B cell receptor sequences to their cognate antigen, HLA-peptide tetramer analysis, and single-cell RNA sequencing of lymphocytes will likely offer greater insights into the immune mechanisms causing PMN leading to the development of more effective and targeted treatments in this challenging disease.

## Author contributions

Conceptualized review topic: EC, SA. Participated in the writing of the paper: EC, YW, KK, MH, HM, GW, LK, BB, DH, SA. Supervision: YW, KK, SA. Each author contributed important intellectual content during manuscript drafting or revision and agrees to be personally accountable for the individual’s own contributions and to ensure that questions pertaining to the accuracy or integrity of any portion of the work, even one in which the author was not directly involved, are appropriately investigated and resolved, including with documentation in the literature if appropriate. All authors contributed to the article and approved the submitted version.

## Funding

EC is supported by the NHMRC Postgraduate Research Scholarship (APP 2014037). GW is supported by the NHMRC Fellowships (APP 1195414 and 1147657). SA is supported by the NHMRC Grants (APP 1183810 and 1161554).

## Conflict of interest

The authors declare that the research was conducted in the absence of any commercial or financial relationships that could be construed as a potential conflict of interest.

## Publisher’s note

All claims expressed in this article are solely those of the authors and do not necessarily represent those of their affiliated organizations, or those of the publisher, the editors and the reviewers. Any product that may be evaluated in this article, or claim that may be made by its manufacturer, is not guaranteed or endorsed by the publisher.

## Glossary

**Table:**

ABBA	Anti brush border antibody
AHN	Active Heymann nephritis
APC	Antigen-presenting cells
AR	Aldose reductase
BAFF	B-cell activating factor
BCR	B cell receptor
Bregs	Regulatory B cells
BSA	Bovine serum albumin
C3aR	Complement 3a receptor
CAAR	Chimeric autoantigen receptor
CAR	Chimeric antigen receptor
CDR-H3	Third complementarity-determining region of the heavy chain
CFH	Complement factor H
CNTN1	Contactin 1
CR1	Complement receptor 1
CTLD	C-type lectin-like domains
DNA	Deoxyribonucleic acid
EXT1/EXT2	exostosin 1/exostosin 2
FAT1	Protocadherin FAT1
FMNL1	Formin-like 1
GBM	Glomerular basement membrane
HLA	Human leukocyte antigen
HTRA1	High temperature requirement A serine peptidase 1
IgG	Immunoglobulin G
IL	Interleukin
LRP2	Low-density lipoprotein receptor-related protein 2
MASP	MBL-associated serine protease
MBL	Mannan-binding lectin
mTOR	Mammalian target of rapamycin
NCAM1	Neural cell adhesion molecule 1
NELL1	Neural epidermal growth factor-like 1 protein
NEP/CD10	Neutral endopeptidase;
NTNG1	Netrin G1
PCDH7	Protocadherin 7
PHN	Passive Heymann nephritis
PLA2R	M-type phospholipase A2 receptor 1
PMN	Primaryn membranous nephropathy
RAG	Recombination activating gene
RAP	Receptor associated protein
Rh-a3NC1	Recombinant human noncollagenous domain 1 of a3(IV) collagen
RNA	Ribonucleic acid
Sema3B	Semaphorin 3B
SLE	Systematic lupus erythematosus
SNP	Single nucleotide polymorphism
SOD2	Superoxide dismutase 2
TCR	T cell receptor
Tfh	T follicular helper
Tfr	T follicular regulatory
TGF-b	Transforming growth factor-b
TGFBR3	Transforming growth factor-b receptor 3
Th	T helper
THSD7A	Thrombospondin type-1 domain-containing protein 7A
TNF	Tumor necrosis factor
Tregs	Regulatory T cells
ULK1	Unc-51 like autophagy activating kinase 1
VDJ	Variable
Diversity	Joining gene segments
